# Genetic variation in released gametes produces genetic diversity in the offspring of the broadcast spawning coral *Acroporatenuis*

**DOI:** 10.1038/s41598-022-08995-3

**Published:** 2022-03-23

**Authors:** Seiya Kitanobo, Sho Toshino, Masaya Morita

**Affiliations:** 1 Sesoko Station, Tropical Biosphere Research Center, University of the Ryukyus, Motobu, Okinawa Japan; 2 Kuroshio Biological Foundation, Kochi, Japan

**Keywords:** Ecology, Zoology

## Abstract

All coral species in the genus *Acropora* are broadcast-spawning hermaphrodites. Fertilization in the ocean requires sufficient numbers of gametes from conspecifics and the contact time for fertilization is thought to be limited by the rapid diffusion of sperm. Many studies have reported a positive correlation between sperm concentration and fertilization success, but it is not clear how gametes diffuse in seawater to produce mixtures of gametes from many colonies, leading to fertilization that improves genetic diversity. To elucidate this, we analyzed the changes in sperm concentration of *A.* *tenuis* in situ after spawning and genotyped sperm and fertilized eggs from seawater using seven microsatellite (MS) markers. Results showed that most of the eggs were fertilized at below 10^6^ sperm/mL in situ. MS genotyping showed that the alleles of released sperm were diverse and those alleles also appeared in the fertilized eggs. The MS fragment peak height in released sperm, which presumably reflects the allele frequency of the sperm, was positively correlated with the allele frequencies of the fertilized eggs. Collectively, synchronous spawning populations composed of highly fecund and genetically diverse colonies potentially increases genetic diversity and the number of descendants.

## Introduction

Sexual reproduction produces genetic diversity in offspring, which enables the selection of genotypes associated with higher fitness and adaptation^1–3^. Reef-building corals in the genus *Acropora* release gametes into seawater synchronously and this broadcast spawning system gives rise to genetic diversity in their offspring; eggs of one colony can potentially mate with sperm from many other colonies.

Climate change and heat wave potentially impact on the reproduction of the coral *Acropora*. Coral reefs are now under threat^4^, and reef degradation from bleaching events has increased^4–6^. Although the remaining corals have a higher thermal threshold for bleaching^7^ and can participate in sexual reproduction, it is plausible that their mating success has declined. The importance of sexual reproduction is noticed, but knowledge of how the process from spawning to fertilization generates genetic diversity in nature (in situ) is still limited.

Many marine invertebrates are benthic or sessile, releasing their gametes into the water column and spawning synchronously to facilitate fertilization^8,9^. Spawning synchronism in marine invertebrates has been widely reported^10–13^, and synchronism is associated with fertilization success^14^.

In broadcast-spawning *Acropora* corals, fertilization success is associated with sperm concentration, which is affected by spawning synchrony, gamete number, and other factors. For example, sympatric *Acropora* species release their gamete bundles synchronously^10^, and in vitro experiments^15,16^ have shown that the fertilization rate depends on sperm concentrations, which in nature are expected to be associated with spawning synchrony^17^, water currents^18^, colony densities^19,20^, and the distance from the sperm source^21^. The timing of spawning and variation in water currents lead to high variation in sperm concentrations that affects the selection of fertilization-related traits. Although data on sperm concentrations in situ after spawning are limited, several studies have examined sperm concentrations after spawning in broadcast spawning corals^22,23^.

When examining sperm concentrations and fertilization success in *Acropora* corals in situ, it can be difficult to isolate one species. However, in Okinawa, Japan, *Acropora* *tenuis* spawns at sunset, about 2.5 h earlier than most other *Acropora* species^24^. Moreover, at Sesoko Island, Okinawa, sunset spawning is dominated by *A*. *tenuis*^25^, making it a good model for investigating the time course from spawning to fertilization.

In this study, we obtained in situ data for *A*. *tenuis* on the time course of sperm concentrations, the genetic diversity of released sperm, and the non-biased fertilization success of released gametes. We discuss the relationship between the genetic diversity of the fertilized eggs that leads to the next generation, and the amount and genetic diversity of the released gametes.

## Materials and methods

### Underwater observation of *Acropora tenuis*

We observed spawning of the scleractinian coral *A*. *tenuis* (n = 11) on a section of the fringing reef of Sesoko Island, Okinawa, Japan (26° 37′ 43.9″ N 127° 51′ 43.3″ E), by snorkeling or SCUBA diving for six nights from May 26 to May 31, 2018. Floats were released from 4 of the 11 tagged colonies (ten15, ten21, ten27, and ten31). The area in which we monitored the floats was approximately 1 × 1 km^2^.

### Sperm concentration in situ

**Figure 1:**
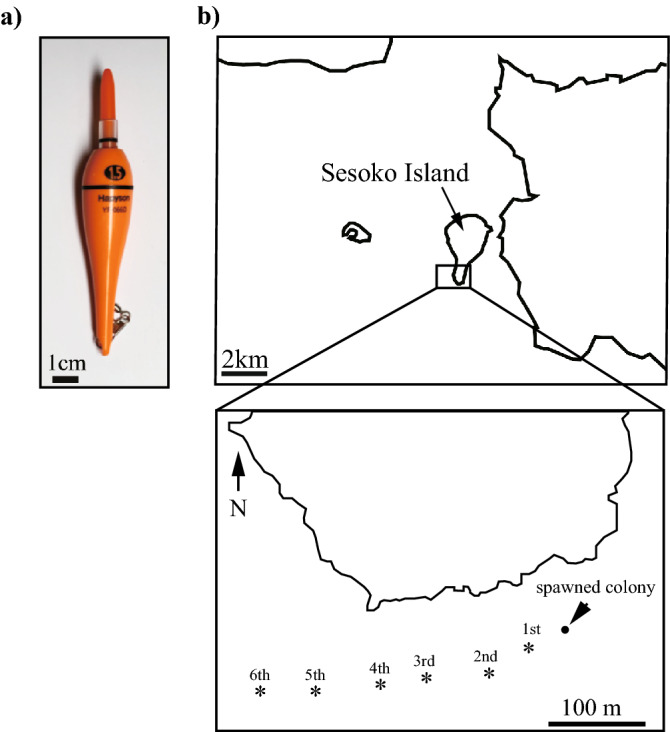
Map of study site. (**a**) Floats used in this study had fluorescent light bars (ϕ 13.0 × 122 mm, Hapyson) attached. (**b**) Trajectory of the float measured in the daytime via GPS. The dot indicates the location of the spawning colony where the float was released. Asterisks indicate the location of water sampling at 9 min intervals.

To trace gamete bundles released from the tagged colonies, floats with fluorescent light bars (Fig. 1a) were released directly above the spawning colonies approximately 10 min after the colonies started spawning. We followed the floats by kayak, and collected 1 L seawater near each float at about 9 min intervals 4 to 6 times (during approximately 1 h) after the release of the floats (Fig. 1b). The collected seawater was brought back to Sesoko Station within 30 min, and the sperm concentrations were measured with a Thoma hemocytometer according to a previous study^12^. The sperm in the collected seawater was measured five times in 200 × 200 µm. When no sperm was found, the concentration was described as being below 2.5 × 10^4^ sperm/mL. It took about 1 h from collecting the seawater to the start of sperm concentration measurement, and by this time most of the eggs in the seawater had already been fertilized.

### Genetic diversity of spermatozoa in seawater and fertilized eggs

The genotypes of sperm and fertilized eggs were determined using microsatellite markers developed for *Acropora*^26^. Eggs in the collected seawater were transferred to fresh 0.22 µm filtered seawater, and fertilized eggs that had completed embryogenesis were fixed with 99.5% ethanol 3 days after collection. The remaining seawater was filtered and sperm were trapped on a membrane (mixed cellulose ester gridded at 0.45 μm, Merck Millipore, MA, USA). The membranes were soaked with 1 mL CHAOS solution (4 M guanidine thiocyanate, 0.1% [v/v] *N*-lauryl sarcosine sodium salt, 0.1 M β-mercaptoethanol, 10 mM Tris–HCl pH 8.0) and the DNA was extracted using a Wizard SV genomic DNA purification system (Promega, WI, USA). DNA was extracted from the fertilized eggs following a previous study with some modifications^12^. Fertilized eggs were kept in filtered seawater for 2 days and fixed in 99.5% EtOH. The fixed larvae were treated with 20 μL f lysis buffer (100 mM NaCl, 10 mM Tris–HCl [pH 8.0], 0.3% [w/v] Triton X-100, 0.3% [w/v] Tween 20) containing 1 g/mL proteinase K for 2.5 h at 55 °C and heated at 95 °C for 5 min. The supernatant was used for PCR reactions for genotyping. As a negative control for sperm detection in seawater, seawater was collected in the daytime following the same protocol. We sampled eight times at 9 min intervals after a float was released.

The sperm in seawater and fertilized eggs were genotyped with seven microsatellite markers with FAM or BIC (12406m3, 11543m5, 11401m4, 441m6, 11292m4, 10366m5, and 12130m5; Supplementary Information 1)^26^. The allele diversity of these seven markers was marked and the fragment amplification was stable. Subsequently, we used the seven markers to verify the presence of the alleles. Fragments were analyzed with a DNA sequencer (Applied Biosystems 3730xl or 3130xl) with GeneScan 500 LIZ dye size standard (Thermo Fisher, MA, USA). Peaks were measured with Microsatellite Analysis v1.0 software (Applied Biosystems by Thermo Fisher, MA, USA). For analyses of seawater containing sperm, peaks below 100 were excluded as alleles (Supplementary Fig. 1). The ratio of each peak in the samples (Rx) was calculated as R_x1_ = (H_x1_/H_1_ + H_2_ + ⋯ + H_x_). Here, H_x1_ indicates the height of one peak among the others, and H_1_ + ⋯ H_x_ is the sum of the heights of all peaks. In the negative seawater control, marker 12406m3 was used and three alleles (176, 179, and 185) were detected in one of the seven samples. These alleles were not detected in sperm in this study (153–174). Thus, the alleles detected in sperm from seawater were treated as those from the released sperm.

### Statistical analyses

The Welch *t*-test was used to examine the differences in allele ratios. To test correlations between the peak height of MS alleles from sperm and the ratio of allele appearance in fertilized eggs, a GLMM was performed with the glmmML package in R ver. 3.1^27^. Each allele was treated as a random effect and the binominal distribution was used as a family.

## Results

### Spawning of tagged colonies

In all, 10 of the 11 tagged *A*. *tenuis* colonies spawned on 29 May and 2 colonies spawned on 30 May. Although we did not record the spawning start times of all colonies, spawning occurred around 19:32 to 19:38 on 29 May and 19:33 to 19:37 on 30 May. Most *A*. *tenuis* colonies released > 1000 gamete bundles each.

### In situ time course of sperm concentration and sperm genetic diversity

**Figure 2:**
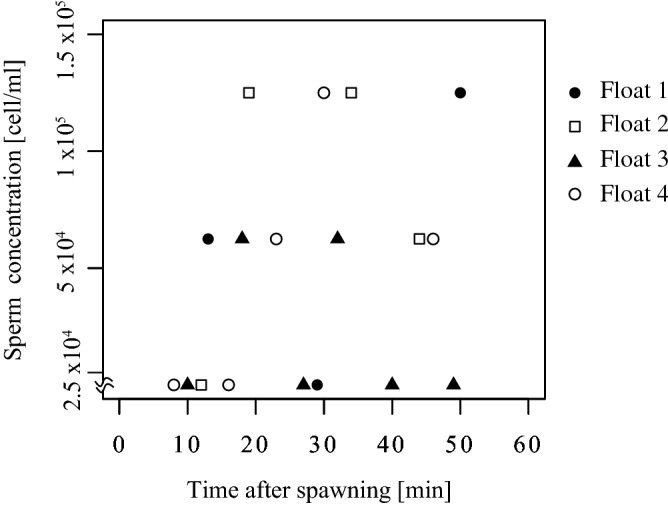
Time course of sperm concentration after the spawning in situ. Sperm concentrations were measured from seawater collected at 9 min intervals after the floats were released. Floats were released from the spawned colony approximately 10 min after they started releasing gametes. Water was collected for six times for Float 1 and four, five times for Float 2, and four times for Float 3.

We followed the floats and collected seawater containing gametes. Most sperm concentrations in the collected seawater were < 10^5^ sperm/mL (Fig. 2). We found no sperm in several samples although microsatellite analyses showed amplification of fragments in seawater in which no sperm were present (see below). The sperm concentrations varied over the course of sampling. Variation in sperm concentrations might have occurred because of differences between water currents and the movement of the floats, which might not represent precise water movements of the gametes from tagged colonies.

**Figure 3:**
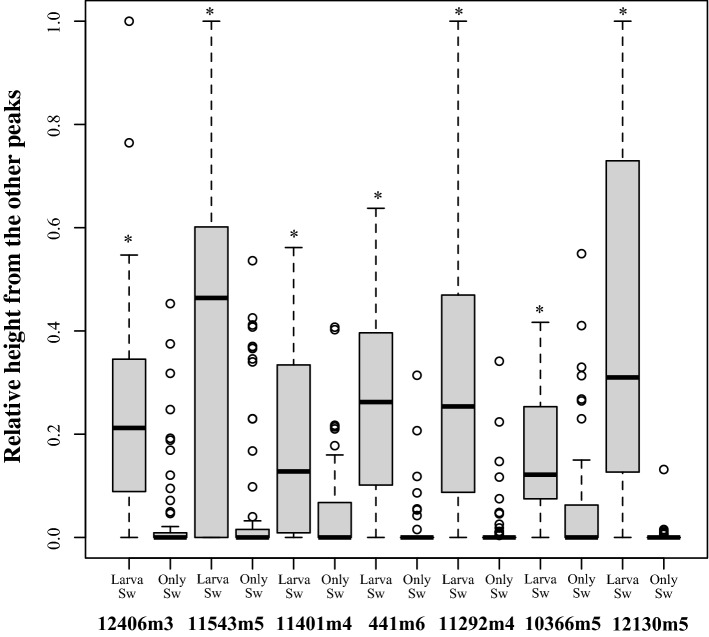
Fragment peaks of alleles. The fragment peaks for each MS marker were compared. Alleles found in sperm/fertilized eggs (Larva/SW) or only in sperm (only SW) are plotted. *P < 0.0001 (Welch t-test, M4 t = 3.95, df = 91.8, M5 t = 4.88, df = 30.5, M7 t = 4.89, df = 47.9, M8 t = 8.39, df = 69.5, M9 t = 7.52, df = 45.0, M12 t = 3.22, df = 85.0, M13 t = 7.71, df = 34.1).

Microsatellite genotyping showed that several alleles were amplified in each seawater sample. The numbers of alleles were indicated by the fragment lengths (bp). The allele numbers varied after spawning (Table 1, Supplementary Information 2). Although sperm were not found in many observations with the hemocytometer, microsatellite fragments were amplified from these samples and the eggs in these samples were fertilized. Most alleles found in sperm were detected in fertilized eggs (Table 1, alleles marked with *). The alleles detected only in seawater had lower MS fragment peak heights in the fragment analyses (Fig. 3).

**Table 1:** Alleles detected in seawater and fertilized egg samples.

Float no.	1st collection	2nd	3rd	4th	5th	6th	Genotypes of spawned colony	Colony no.
**12406m3**								
1	156^¶^, 159*^,†^, 162, 165, 168 (1 egg)	156, 159^†^, 162, 165* (2 eggs)	156, 159*^,†^, 162, 165* (6 eggs)	156*, 159*^,†^, 162*, 165* (31 eggs)	156*, 159*^,†^, 162*, 165* (24 eggs)	156*, 159*^,†^, 162*, 165*, 171^¶^, 174 (25 eggs)	159, 171	ten15
2	156, 159*, 162*, 165* (9 eggs)	153, 159* (2 eggs)	156^¶^, 159*, 162^¶^, 165* (9 eggs)	153, 156^¶^, 159*, 162*, 165*, 168^¶^ (6 eggs)	153*, 156*, 159*, 162*, 165*, 168*, 174 (117 eggs)	Seawater was not collected	162	ten21
3	159*, 162^¶^, 165^¶^ (2 eggs)	N.D. (2 eggs)	156*, 159*, 162*, 165*, 168 (47 eggs)	156*, 159*, 162*, 165*, 168*, 171, 174 (96 eggs)	Seawater was not collected	Seawater was not collected	194	ten27
4	156, 159, 162, 165^†^, 168	N.D	156, 159, 162, 165^†^	165^†^	156, 159, 162, 165^†^	156, 159, 162, 165^†^, 168, 171	153, 165	ten31
**11543m5**								
1	126*	120, 122, 126*, 130	120, 122, 126*, 130, 132^¶^	120, 122, 126*, 130, 132^¶^	120, 122, 126*, 130, 132^¶^	120, 122, 126*, 130, 132^¶^	128	ten15
2	120, 122, 126*, 128^†^, 130, 132^¶^	120, 122, 126*, 130, 154	126*, 130, 132^¶^	126*, 130, 132^¶^	120, 122, 126*, 128^†^, 130, 132^¶^	Seawater was not collected	128.132	ten21
3	126*, 130, 132^¶^	126*, 130, 132^¶^	120, 122, 126*, 130, 132^¶^	120, 122, 126*, 128^†^, 130, 132^¶^	Seawater was not collected	Seawater was not collected	1,28,132	ten27
4	120, 122, 124, 126, 128^†^, 130, 154	126, 130	126, 128^†^, 130	126, 130	126, 130	126, 130, 230	128, 132	ten31
**11401m4**								
1	403*, 407^¶^, 411, 415	391*, 399, 410, 403*, 407, 411	399, 401, 403*, 407	399*, 403*, 407*, 411	391*, 395, 399*, 401^¶^, 403*, 407*, 411	391*, 399*, 403*, 407*, 411	N.D	ten15
2	391, 395, 399^†^, 403*, 407*, 411	391*, 395, 399^†^, 401, 403*, 407, 411	395^¶^, 399*^,†^, 403, 405^¶^, 407*	391, 399*^,†^, 401^¶^, 403*, 407^¶^, 409, 411	391*, 395, 399*^,†^, 401*, 403*, 405, 407*, 409, 411	Seawater was not collected	399	ten21
3	383, 399, 403*, 407*, 411,415	383, 391, 399*, 401^¶^, 403*, 407*	391*, 395^¶^, 399*, 403*, 407*, 409^¶^, 411	391*, 399*, 401^¶^, 403*, 407*, 411	Seawater was not collected	Seawater was not collected	409, 413	ten27
4	383, 395, 399, 403, 407, 411	403	403	391, 403	399, 407	391, 395, 401, 403, 407, 409^†^, 411	409, 413	ten31
**441m6**								
1	285*, 291*	285*, 291*, 297*,303	285*, 291*, 297*, 303	285*, 291*,297*, 299^¶,†^, 303^¶^	285*, 291*, 297*, 299^¶^, 303	285*, 291*, 297*, 303*	299	ten15
2	285*, 291*, 297, 299*^,†^	279, 285*, 291*, 297	285*, 291*, 297*, 303	285*, 291*^,†^, 297*, 303	285*, 291*^,†^, 297*, 299^¶^, 303*	Seawater was not collected	291	ten21
3	285*^,†^, 291*	285*^,†^, 291*, 297^¶^, 299^¶^	285*^,†^, 291*, 297*, 299^¶^	279^¶^, 285*^,†^, 291*, 297*, 299^¶^, 303	Seawater was not collected	Seawater was not collected	285	ten27
4	285, 292, 297^†^, 303	N.D	285, 292, 297^†^	285, 291, 303	N.D	285, 291, 297^†^	297	ten31
**11292m4**								
1	471*, 483*, 487	471*, 475, 483, 487*, 495	471*, 475, 483, 487*	471*, 475, 483*, 487*, 495	471*, 475*, 479, 483*, 487*, 495	471*, 475, 483*, 487*	N.D	ten15
2	471*^,†^, 475*, 483*, 487^¶^, 491^¶^	471*^,†^, 483^¶^	471*^,†^, 475, 483*, 487	463^¶^, 471*^,†^, 483*, 487, 491	463*, 471*^,†^, 483*, 487*, 491*	Seawater was not collected	471	ten21
3	471*, 475^¶^, 483^†^	N.D	471*, 475, 483*^,†^, 487*	471*, 479, 483*^,†^, 487*, 491*	Seawater was not collected	Seawater was not collected	483	ten27
4	471^†^, 483^†^, 487	N.D	471^†^, 475, 483^†^, 487	N.D	471^†^	471^†^, 483^†^	471, 483	ten31
**10366m5**								
1	215* 217, 223^†^, 225, 227^¶^	215*, 217, 223^†^, 225	215*, 217^¶^, 223^¶^, 225*, 227^¶^	213*, 215*, 217*, 225*, 227*	213*, 215*, 217*, 225*, 227*	213*, 215*, 217*, 223^¶^, 225*, 227	223	ten15
2	213*, 215*, 217^¶^, 223^¶^, 225*, 227	N.D	N.D	211^¶^, 215*, 217^¶^, 223^†^, 225*	209^¶^, 213*, 215*, 217*, 219, 223*^,†^, 225*, 227	Seawater was not collected	223	ten21
3	N.D	N.D	209*, 211, 213*, 215*, 217*^,†^, 225*, 227*	213*, 215*^,†^, 217*^,†^, 219, 223*, 225*, 227*	Seawater was not collected	Seawater was not collected	217	ten27
4	N.D	N.D	N.D	N.D	N.D	N.D	223	ten31
**12130m5**								
1	241*^,†^, 277*	241*^,†^, 277*	241*^,†^, 269*, 277*	241*^,†^, 261, 277*	241*^,†^, 261^†^, 277*	241*, 269*, 277*	241, 261	ten15
2	241*^,†^, 277*^,†^	241*^,†^, 277^¶,†^	241*^,†^, 269^¶^, 277*^,†^	241*^,†^, 269*, 277^†^	241*^,†^, 261, 269*, 277*^,†^	Seawater was not collected	241, 277	ten21
3	241*^,†^, 277*	241*^,†^, 277*	241*^,†^, 277*	229, 241*^,†^, 261, 269*, 277*	Seawater was not collected	Seawater was not collected	241	ten27
4	241^†^, 277	215, 241^†^	241^†^, 277	241^†^, 277	241^†^, 277	241^†^, 277	241	ten31

*Alles found in both fertilized eggs and sperm, ^¶^Alleles found only in fertilzed eggs, ^†^Alleles from tagged colony, alleles without mark was found only in sperm.

### Genetic diversity of sperm and fertilized eggs in seawater

When genotyping the sperm samples, many alleles were detected for each marker, and the numbers varied by MS markers, sperm, and fertilized eggs (Table 1, Supplementary Information 2 and 3). We released floats from the spawning colonies to follow released gametes, but the alleles found in the collected seawater often did not match those of the spawned colony (Table alleles from tagged colonies are marked with †).

We examined 1 to 60 fertilized eggs for floats 1 to 3, but we could not analyze eggs found for float 4 due to loss of the samples. Many alleles were detected and most of these matched those in the same seawater sample. The alleles present changed over the sampling time (Table 1, Supplementary Information 3). Several alleles in sperm samples were not detected in the fertilized eggs (Table 1, alleles are not marked), and there were alleles that found only in the fertilized eggs (Table 1, alleles marked with ¶).

**Figure 4:**
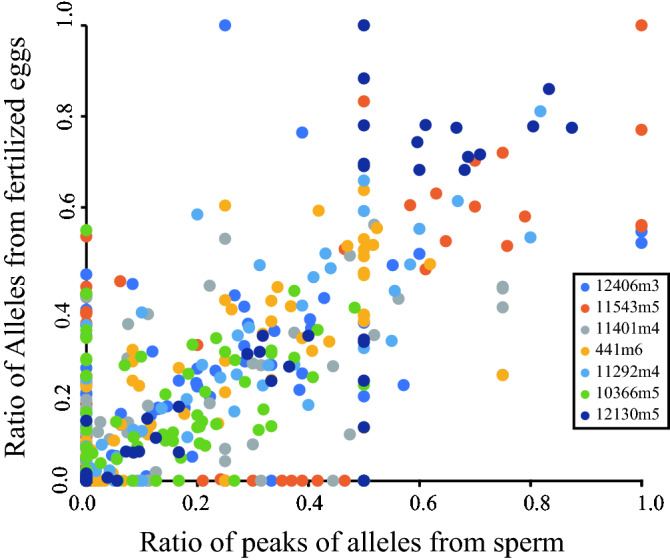
Relationship between alleles in the fertilized eggs and sperm in situ. Ratio of the numbers of the each MS allele (e.g., 191) and those of all MS alleles (e.g., 191 and 197) in the fertilized eggs in each seawater sample (vertical line) (e.g., Allele 191 in Float1, third collection is 2/12 = 0.167 Supplementary Information 1). The horizontal line indicates the ratio of peak heights in fragment analyses of each seawater sample (e.g., ratio of allele 191 in Float 1, third collection was = 0.16). Alleles of each MS in this study are indicated by different symbols.

When comparing the fragment peaks of the alleles, MS fragment peak height was positively correlated with the frequency of the alleles in the fertilized eggs (Figs. 3 and 4, Supplementary Information 2 and 3). Several alleles were detected only in sperm samples and these had smaller peaks (Fig. 3, Supplementary Information 2 and 3). However, we could not distinguish which alleles in the fertilized eggs were derived from sperm or eggs. Alleles in the fertilized eggs were positively correlated with the peak heights in the fragment analyses (GLMM *P* < 0.001, coefficient = 6.9; Fig. 4).

## Discussion

The genetic diversity represented by allele frequencies in fertilized eggs was correlated with the genetic diversity of released sperm. After spawning, the sperm concentrations in situ varied and were often lower (< 10^5^ sperm/mL) than the “ideal” level (10^6^ sperm/mL)^15,16^, but fertilization was accomplished in situ. Microsatellite analyses showed that many alleles of the fertilized eggs matched those of sperm in situ. In addition, the frequencies of the alleles in fertilized eggs were positively correlated with MS fragment peak heights, which corresponds to the amount of sperm with those alleles. Therefore, mating success in reef-building *Acropora* depends on the amount of released sperm in situ, concurring with many previous in vitro fertilization trials; more sperm have greater chances of successfully fertilizing eggs.

The sperm concentration in situ was lower than we expected, but most eggs in the samples were fertilized. It took at least 1.5 h to examine egg fertilization. Many studies have indicated that fertilization in vitro decreases below 10^5^ sperm/ML^15,16,28^. The sperm and eggs were kept in containers after collection, and so the condition for fertilization is not precisely represented in situ. The fertilization ratio is often dependent on the combination of colonies. For example, the fertilization rates of *A*. *tenuis* around Sesoko Station vary according to the combination of gametes from conspecific colonies; several combinations of colonies have very low fertilization rates^25^. While this needs to be examined, the alleles found in the fertilized eggs were correlated with the peak height of each allele, which is representative of sperm numbers.

Although the fertilization ratio in situ was potentially overestimated, fertilization might be accomplished soon after sperm and eggs are mixed^29^. Moreover, sperm–egg interactions due to chemoattractants may contribute to their successful fertilization at lower sperm concentrations^30^. If sperm can efficiently interact with unfertilized eggs with the assistance of chemoattractants and fertilization finishes quickly, fertilization with many different sperm from many colonies may occur, even at low sperm concentrations. However, we have no practical observations or information about gamete interactions, such as details of the release of bundles from colonies, bundles separated into sperm and eggs, and diffusion of these gametes.

The genetic inheritance of fertilized eggs, number of alleles, and their frequencies were positively correlated with sperm alleles found in the water. Most alleles found in the fertilized eggs were also found in the seawater containing sperm (Table 1, alleles marked with *). Presumably, most alleles found in both seawater and eggs represent the alleles in the fertilizing sperm, but several alleles were found only in the fertilized eggs (Table 1, alleles marked with ¶) and these alleles may be from both sperm and eggs. Although we could not distinguish the origin of alleles (sperm or eggs), the MS fragment peak heights of alleles found in seawater and fertilized eggs were slightly higher than those of alleles found only in seawater (Fig. 3), suggesting that the alleles found in more sperm in seawater completed fertilization in situ. There is almost no information on which alleles are passed to the next generation in corals.

Genetic inheritance in *Acropora* needs more study regarding the fertilization of gametes according to sperm concentration. In a previous study, eggs preferred conspecific sperm, while the proportion of fertilization by heterospecific sperm increased at lower sperm concentrations^12^. This implies that fertilization matching conspecific sperm and eggs is complicated and lower sperm concentrations might be associated with hybridization. In this study, we collected gametes in the ocean off Sesoko Island to follow the fertilization process in *A*. *tenuis*. Near this island, *A*. *donei*, which can potentially mate with *A*. *tenuis*, does not release a large number of eggs^25^. In addition, *A*. *tenuis* sperm does not fertilize eggs of *A*. *donei* in the presence of conspecifics^24,25^. Therefore, hybridization between *A*. *tenuis* and *A*. *donei* rarely occurs. We need to consider the breeding success of conspecifics or hybridization in later-spawning intercrossing species such as *A*. *intermedia* and *A*. *florida*. Fertilization in situ among intercrossing species may be associated with hybridization, which could be associated with adaptation to climate change^31^. Future studies need to address this.

## Supplementary Information

Supplementary Information 1.

Supplementary Information 2.

Supplementary Information 3.

Supplementary Information 4.
